# Supplementary material for: Prevalence, trend and associated factors of obesity-related cancers among U.S. adults with metabolic syndrome: Evidence from the National Health and Nutrition Examination Survey 2001–2018
Source: PLoS One. 2023 Sep 1;18(9):e0290994. doi: 10.1371/journal.pone.0290994 (PMC10473473; doi:10.1371/journal.pone.0290994)
Supplement: S1 Checklist — (DOCX) [file pone.0290994.s001.docx]

STROBE Statement—checklist of items that should be included in reports of observational studies

|  | Item No. | Recommendation | Page  No. | Relevant text from manuscript |
| --- | --- | --- | --- | --- |
| **Title and abstract** | 1 | (*a*) Indicate the study’s design with a commonly used term in the title or the abstract | 1 | This study used cross-sectional data from the 2001-2018 National Health and Nutrition Examination Survey |
|  |  | (*b*) Provide in the abstract an informative and balanced summary of what was done and what was found | 1-2 | The ORC prevalence was 35.8% representing 4463614 adults with MetS. A higher likelihood of ORC was observed among females (OR=7.1, CI=4.9-10.3) vs males, Hispanic (OR=2.9, CI=1.7-4.8)…. |
| Introduction | | | |  |
| Background/rationale | 2 | Explain the scientific background and rationale for the investigation being reported | 2-4 | Obesity and cancer are two interlinked major public health issues in the United States and globally (1,2). Overall cancer is the second leading cause of death in the United States (3), and nearly 40% of all cancer diagnoses in the U.S. are obesity-related cancer (ORC)… |
| Objectives | 3 | State specific objectives, including any prespecified hypotheses | 4 | This study aims to determine the prevalence and predictors of ORC based on recent data, and further evaluate the temporal trends in ORC among U.S. adults with comorbid conditions MetS and cancer… |
| Methods | | | |  |
| Study design | 4 | Present key elements of study design early in the paper | 4 | Data were collected from National Health and Nutrition Examination Survey (NHANES) 2001-2018. NHANES uses a complex, multistage, probability sampling design to collect a representative sample of the non-institutionalized U.S. population. NHANES collects data in two-years cycles… |
| Setting | 5 | Describe the setting, locations, and relevant dates, including periods of recruitment, exposure, follow-up, and data collection | 4 | Data were collected from National Health and Nutrition Examination Survey (NHANES) 2001-2018. NHANES uses the complex survey design to collect a representative sample of the U.S. population…. |
| Participants | 6 | (*a*) *Cohort study*—Give the eligibility criteria, and the sources and methods of selection of participants. Describe methods of follow-up  *Case-control study*—Give the eligibility criteria, and the sources and methods of case ascertainment and control selection. Give the rationale for the choice of cases and controls  *Cross-sectional study*—Give the eligibility criteria, and the sources and methods of selection of participants | 4-5 | Adults of age 20 and over with diagnoses of MetS and cancer were included in this study. Pregnant women were excluded from the study as they tend to have temporary MetS. |
|  |  | (*b*) *Cohort study*—For matched studies, give matching criteria and number of exposed and unexposed  *Case-control study*—For matched studies, give matching criteria and the number of controls per case |  |  |
| Variables | 7 | Clearly define all outcomes, exposures, predictors, potential confounders, and effect modifiers. Give diagnostic criteria, if applicable | 5 | The respondent was labeled as having an obesity related Cancer (ORC) even if the other two cancers were not obesity-related. Predictors included: age (20-39, 40-59 and 60+), gender (male, female), race (Hispanic, non-Hispanic white, non-Hispanic black), education (high school graduate or less, some college degree, some college or above), annual household income ($35,000, $35,000 to $74,999, or $75,000+), country of birth (US-born, Mexico-born, and others), insurance status (yes, no), physical activity (yes: moderate or vigorous activity, no: otherwise), smoking (never, former, current), and alcohol use (never, former-drinker, mild, and heavy-drinker). |
| Data sources/ measurement | 8* | For each variable of interest, give sources of data and details of methods of assessment (measurement). Describe comparability of assessment methods if there is more than one group | *4-5* | *Data were collected from National Health and Nutrition Examination Survey (NHANES) 2001-2018. NHANES uses the complex survey design to collect a representative sample of the U.S. population* |
| Bias | 9 | Describe any efforts to address potential sources of bias | NA |  |
| Study size | 10 | Explain how the study size was arrived at | 4 | Data from 9 NHANES periods were combined to obtain a total period 2001-2018 … |

Continued on next page

| Quantitative variables | 11 | Explain how quantitative variables were handled in the analyses. If applicable, describe which groupings were chosen and why | 5 | Predictors included: age (20-39, 40-59 and 60+), gender (male, female), race (Hispanic, non-Hispanic white, non-Hispanic black), education (high school graduate or less, some college degree, some college or above), annual household income ($35,000, $35,000 to $74,999, or $75,000+), country of birth (US-born, Mexico-born, and others), insurance status (yes, no), physical activity (yes: moderate or vigorous activity, no: otherwise), smoking (never, former, current), and alcohol use (never, former-drinker, mild, and heavy-drinker). |
| --- | --- | --- | --- | --- |
| Statistical methods | 12 | (*a*) Describe all statistical methods, including those used to control for confounding | 6 | All analyses in this study were adjusted by appropriate sampling weights (fasting sampling weights) to ensure nationally representative estimates (29). The analyses were conducted in R using survey package… |
|  |  | (*b*) Describe any methods used to examine subgroups and interactions | 6 | The prevalence of ORC for sociodemographic and behavioral characteristics was calculated. The Rao-Scott chi-square tests were performed to determine differences in these characteristics between ORC and non-ORC for categorical predictors and the t-test for a continuous predictor. … |
|  |  | (*c*) Explain how missing data were addressed | 5 | Note that participants that were selected to give a fasting blood sample constructed the smallest survey subsample, and appropriate probability sampling weights were calculated by NHANES to make it representative of the U.S. population… |
|  |  | (*d*) *Cohort study*—If applicable, explain how loss to follow-up was addressed  *Case-control study*—If applicable, explain how matching of cases and controls was addressed  *Cross-sectional study*—If applicable, describe analytical methods taking account of sampling strategy | 5 | All analyses in this study were adjusted by appropriate sampling weights (fasting sampling weights) to ensure nationally representative estimates… |
|  |  | (*e*) Describe any sensitivity analyses | NA |  |
| Results | | | | |
| Participants | 13* | (a) Report numbers of individuals at each stage of study—eg numbers potentially eligible, examined for eligibility, confirmed eligible, included in the study, completing follow-up, and analysed | 7 | In the total study period 2001-2018, a total of 91351 participants were screened for eligibility. After removing incomplete data (who were not selected to give a fasting blood sample), 30065 participants were found to be eligible initially… |
|  |  | (b) Give reasons for non-participation at each stage | 7 | After removing incomplete data (who were not selected to give a fasting blood sample), 30065 participants were found to be eligible initially… |
|  |  | (c) Consider use of a flow diagram | 7 | Fig 1 |
| Descriptive data | 14* | (a) Give characteristics of study participants (eg demographic, clinical, social) and information on exposures and potential confounders | 7-8 | Table 1 shows the number of participants and the prevalence of ORC, overall and within each subgroup, during the total study period 2001-2018…… |
|  |  | (b) Indicate number of participants with missing data for each variable of interest | NA |  |
|  |  | (c) *Cohort study*—Summarise follow-up time (eg, average and total amount) |  |  |
| Outcome data | 15* | *Cohort study*—Report numbers of outcome events or summary measures over time |  |  |
|  |  | *Case-control study—*Report numbers in each exposure category, or summary measures of exposure |  |  |
|  |  | *Cross-sectional study—*Report numbers of outcome events or summary measures | *7* | *Table 1 shows the number of participants and the prevalence of ORC, overall and within each subgroup, during the total study period 2001-2018* |
| Main results | 16 | (*a*) Give unadjusted estimates and, if applicable, confounder-adjusted estimates and their precision (eg, 95% confidence interval). Make clear which confounders were adjusted for and why they were included | 7-13 | Table 1 shows the number of participants and the prevalence of ORC, overall and within each subgroup, during the total study period 2001-2018…. |
|  |  | (*b*) Report category boundaries when continuous variables were categorized | 7-13 | Table 1, 2 and 3 |
|  |  | (*c*) If relevant, consider translating estimates of relative risk into absolute risk for a meaningful time period |  |  |

Continued on next page

| Other analyses | 17 | Report other analyses done—eg analyses of subgroups and interactions, and sensitivity analyses | 11 | The overall age-adjusted ORC prevalence in all three cohorts remained stable (Table 3). But the age-standardized ORC prevalence increased gradually among females, increasing from 40.44% in 2001-2006 to 46.35% in 2013-2018, while the prevalence decreased for males from 13.73% in 2001-2006 to 8.17% in 2013-2018….. |
| --- | --- | --- | --- | --- |
| Discussion | | | | |
| Key results | 18 | Summarise key results with reference to study objectives | 13-15 | According to findings, the occurrence of ORC was more common in non-Hispanic Black and Hispanic participants with MetS, with a prevalence of over 50% for each group, as compared to non-Hispanic Whites with MetS…. |
| Limitations | 19 | Discuss limitations of the study, taking into account sources of potential bias or imprecision. Discuss both direction and magnitude of any potential bias | 16 | The limitation of this study is that it used cross-sectional data, which means it cannot establish the causality of the observed associations…. |
| Interpretation | 20 | Give a cautious overall interpretation of results considering objectives, limitations, multiplicity of analyses, results from similar studies, and other relevant evidence | 16 | This study offers valuable insights into the prevalence and temporal trends of ORC among U.S. adults with MetS. The higher prevalence of ORC among females, Hispanics, and non-Hispanic blacks suggests that targeted interventions may be required for these groups to prevent ORC …. |
| Generalisability | 21 | Discuss the generalisability (external validity) of the study results | 16 | Overall, this study underscores the importance of addressing MetS and lifestyle factors to prevent ORCs among U.S. adults with MetS. |
| Other information | |  | | |
| Funding | 22 | Give the source of funding and the role of the funders for the present study and, if applicable, for the original study on which the present article is based | 16 | The author(s) received no specific funding for this work. |
|  |  |  |  |  |

*Give information separately for cases and controls in case-control studies and, if applicable, for exposed and unexposed groups in cohort and cross-sectional studies.

**Note:** An Explanation and Elaboration article discusses each checklist item and gives methodological background and published examples of transparent reporting. The STROBE checklist is best used in conjunction with this article (freely available on the Web sites of PLoS Medicine at http://www.plosmedicine.org/, Annals of Internal Medicine at http://www.annals.org/, and Epidemiology at http://www.epidem.com/). Information on the STROBE Initiative is available at www.strobe-statement.org.
