# Supplementary material for: Prevalence, trend and associated factors of obesity-related cancers among U.S. adults with metabolic syndrome: Evidence from the National Health and Nutrition Examination Survey 2001–2018
Source: PLoS One. 2023 Sep 1;18(9):e0290994. doi: 10.1371/journal.pone.0290994 (PMC10473473; doi:10.1371/journal.pone.0290994)
Supplement: Attachment — Submitted filename: authors.pdf [file pone.0290994.s004.pdf]

To the authors:

The manuscript states as the objective to estimate the prevalence of obesity related cancer (ORC) in the US population, trends of such cancers and risk factors for ORC in the US adult population with metabolic syndrome (MetS). The definition of ORC is based on a cancer being of a certain type, identified as being obesity related. The authors provide a definition of MetS based on certain metabolic measurements. The data used to investigate the objectives are from the NHANES surveys conducted from 2001 through 2018 and are de=identified and publicly available.

The authors included all participants of NHANES for those years who obtained a fasting plasma glucose level, were not pregnant and had a history of cancer. A person was classified as having ORC if at least one cancer diagnosed was in the obesity related category. The primary outcome studied was ORC vs non-ORC where non-ORC is a cancer not in the group of cancers defined as ORC. Time trends were investigated by dividing the time period 2001-2018 into three segments: 2001-2006, 2007-2012 and 2013-2018. The authors refer to these subgroups as cohorts. The risk factors studied were: gender, race, age divided into three groups, education, income divided into three levels, country of birth, health insurance, physical activity, smoking history and alcohol use.

The statistical methods used consisted of bivariate analyses with the one variable being ORC vs non ORC, and the other variable one of the risk factors. Furthermore, a multivariable logistic regression was used to investigate the effect of several variables simultaneously on being diagnosed with an ORC. All analyses were conducted using sample survey methodology to adjust for the sampling weights and obtain rates for the US population.

There are several issues with the approach chosen by the authors. First, only subjects with a cancer diagnosis were selected. If this subset differs substantially from the subset of the population that is free of cancer it is possible to miss a risk factor that is specifically related to ORC but not a risk factor for cancer in general. The design chosen by the authors allows only the identification of factors relative to a ORC vs non-ORC cancer diagnosis. It is not clear that the weights calculated in the NHANES survey adjust for the population diagnosed with cancer. If that is the case, this needs to be stated explicitly. If not, then the weights used do not project onto the US population but only onto the cancer population. Thus, it Is not possible to calculate a prevalence of ORC but only to determine the proportion of ORC’s among all the cancers.

The bivariate analyses, chi-squared tests chosen by the authors should only serve as a selection for relevant variables in the logistic regression model. Since the cancer cases are not a random subset of all surveyed subjects, it is not clear that the bivariate parameters have any interpretation as marginal parameters for the US population. For risk factor assessment, as the authors state, causal statements are not possible, therefore, any factor potentially related to the type of cancer should be stated as being associated with ORC or not. Furthermore, other variables such as age that are likely confounders, need to be adjusted for.

The term cohort for the three groups of years is inappropriate. A cohort is a group that is selected and then observed forward. This is not the case here. There are 18 separate samples, one for each year. It is questionable to assess trends in any meaningful way over time since risk due to such factors as age, year born etc are confounded with time trends. Also, it does not seem that the authors directly tested trends over time except to see if cohort as the groupings are named is a predictor of outcome.

There do not seem to be any time trends and the total number of cancers is more or less constant. Therefore, if some types of cancers increase, others must decrease. It comes as no surprise then that opposing trends are seen in different subpopulations. All analyses here must be seen in the context that the overall numbers seem to be fairly constant except perhaps, a bit lower during the first time grouping.

Also, it should be stated somewhere in the paper why the cancers chosen are called ORC. This is not obvious and obviously, not every breast or uterine cancer or any other cancer is due to obesity. Is it even known what proportion of these cancers are attributable in the population to obesity? Why was BMI or a similar measure not used as a covariate?

Many of the cancers are age-related and it is not clear to what degree these ORC are due to age and to what degree due to obesity. If a person is not overweight and does not have metabolic syndrome, then whatever cancer they have, obesity is not the cause. If the purpose is to assess the degree to which obesity and metabolic syndrome contribute to cancer burden, it should be studied in the population that includes non-cancer cases as well.

Some specific remarks/corrections:

Line 69: and reduced likelihood…screening and difficulties… the authors refer to 2 different issues.
Line 72: ninimize the risk
Line 82: two-year cycle
Line 95/96: elevated triglycerides stated twice
Line 129: reference for direct method of standardization
Line 132: what Z-test was used?
Line 133: was time included as year beginning with either 2001 or 1? Also, it should be stated that a linear trend was assessed on the logit scale.
Line 170: omit THE and start the sentence with Multivariable….
Line 171: found to be..
Line 172: higher than in males
Line 195: stable for the three cohorts
Line 233 from multivariable logistic regression
Line 265: MetS with thyroid….cancers being the most…
Line 271: MetS, which maybe attributable to unless it is known to be the case.
