# Supplementary material for: Prevalence, trend and associated factors of obesity-related cancers among U.S. adults with metabolic syndrome: Evidence from the National Health and Nutrition Examination Survey 2001–2018
Source: PLoS One. 2023 Sep 1;18(9):e0290994. doi: 10.1371/journal.pone.0290994 (PMC10473473; doi:10.1371/journal.pone.0290994)
Supplement: Attachment — Submitted filename: Review report- MN.docx [file pone.0290994.s002.docx]

**Title:** Prevalence, trend and risk factors of obesity-related cancers among U.S. adults with metabolic syndrome: Evidence from the National Health and Nutrition Examination Survey 2001-2018

This study is about Prevalence, trend and risk factors of obesity-related cancers among U.S. adults with metabolic syndrome. The importance of this study is vague despite a similar study entitled ““Prevalence and predictors of obesity-related cancers among racial/ethnic groups with metabolic syndrome”, which was published in PLOS one journal”. By the way, I reviewed the paper and there are some comments as follows:

**Abstract:**

- Introduction: Please use related factors instead of predictors.

-in the results section, please identify the level of confidence for CI, i.e. (OR=7.1, 95% CI=4.9-10.3). “However, an increasing trend was seen for thyroid, uterine, colorectal and ovarian cancers while decreasing trend for breast cancer” . P value?

**Introduction:**

- The necessity and importance of this study is not clear. Although, you cited to a similar paper entitled “Prevalence and predictors of obesity-related cancers among racial/ethnic groups with metabolic syndrome”, which was published in PLOS one journal. But, you did not provide information about the difference between this study and yours. I know that the year of evaluation is different, and concentrated on ethnic groups. But, you could not justify the reason of conducting, which is necessary to notice that.
- “. This study aims to determine the prevalence and predictors of ORC based on recent data, and further evaluate the temporal trends in ORC 74 among U.S. adults with comorbid conditions MetS and cancer.” Please use past tense (e.g. This study aimed to …)
- This study is a cross-sectional study, which is differ from prediction model studies. Please use related factors instead of predictors. Because, the aim of this study is not to draw a prediction model and evaluate its performance. Thus, please revise it in the whole manuscript.

**Methods:**

- It is better to write this section according to STROBE guideline.
- There is no information on how to do a univariate logistic regression.

**Results:**

- Please report mean (SD) instead of mean (SE) in the table 1.
- How about univariate logistic regression analysis?

**Discussion:**

- **Line 235-236: “**Additionally, we observed that Hispanic and non-Hispanic black 236 individuals displayed elevated likelihood in comparison to non-Hispanic white”. Likelihood is not an appropriate term to use. You provide odds ratio measure. It is better to use odds instead of likelihood. If the out come prevalence is less than 5, you can use risk interchangeably. Please revise it in the whole document.
- **Line 235- 239: “**Additionally, we observed that Hispanic and non-Hispanic black individuals displayed elevated likelihood in comparison to non-Hispanic whites. Similarly, Monestime et al. (6) found higher odds of ORC among Hispanic and non-Hispanic black individuals with MetS compared to non-Hispanic individuals with MetS, although they did not control behavioral factors, physical activity, and alcohol use”. Adjusting for other confounders can not be the main reason to conduct another study.

**Study limitation:**

- The authors focused on the study strength, which can be mentioned while discussing. But, I think the major limitation is that investigator depended on the existing variables and such variables such as stress, quality of life and other factors was not considered. This section is very important, because it helps author researcher to design new studies.

**Conclusion:**

- In conclusion section, pleas not use reference. In this section, the authors should provide a conclusion on their own words.
